# Supplementary material for: Heterozygous and generalist MxA super-restrictors overcome breadth-specificity trade-offs in antiviral restriction
Source: Sci Adv. 2025 May 2;11(18):eadu0062. doi: 10.1126/sciadv.adu0062 (PMC12047444; doi:10.1126/sciadv.adu0062)
Supplement: Supplementary file 1 — Figs. S1 to S4 Legends for tables S1 to S15 Legend for data S1 [file sciadv.adu0062_sm.pdf]

Science Advances
AAAS

Supplementary Materials for

Heterozygous and generalist MxA super-restrictors overcome breadth-specificity trade-offs in antiviral restriction

Rechel A. Geiger *et al.*

Corresponding author: Harmit S. Malik, hsmalik@fredhutch.org

Sci. Adv. **11**, eadu0062 (2025)
DOI: 10.1126/sciadv.adu0062

The PDF file includes:

Figs. S1 to S4
Legends for tables S1 to S15
Legend for data S1

Other Supplementary Material for this manuscript includes the following:

Tables S1 to S15
Data S1

Fig. S1.

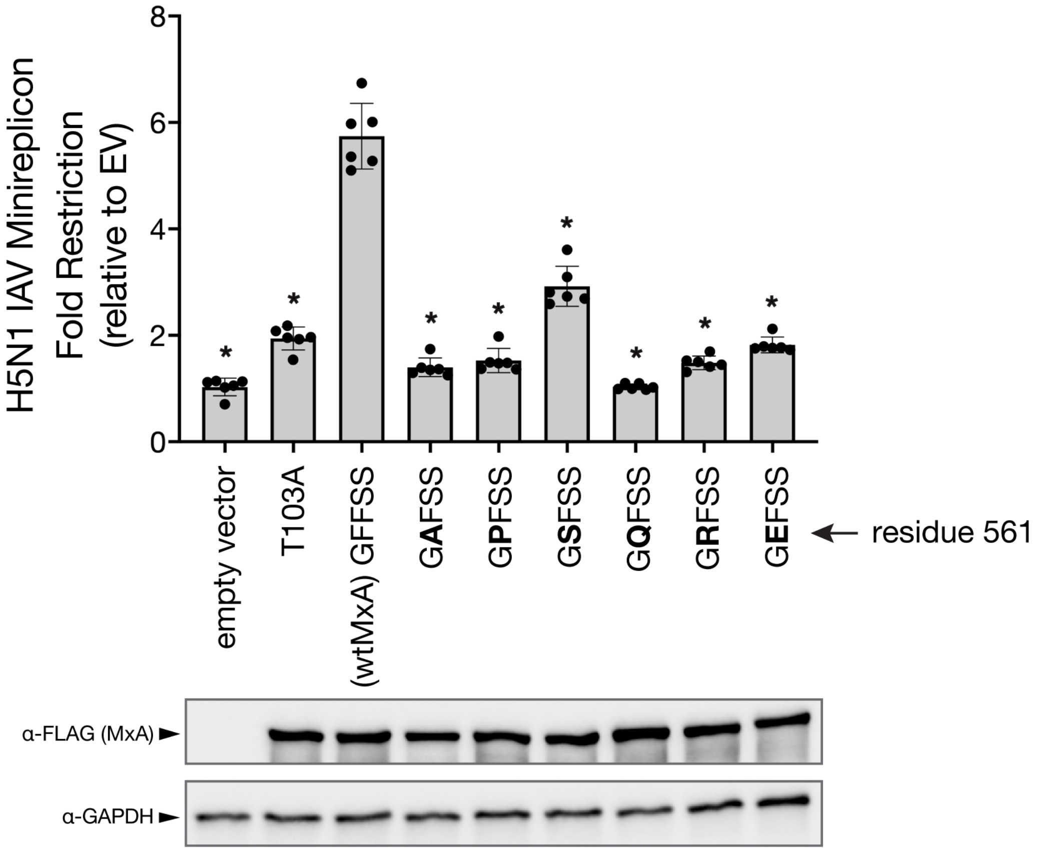

Figure S1: Restriction profiles of MxA variants with various non-aromatic amino acid residues at position 561. Fold restriction is reported relative to an empty vector. Each variant is labeled using amino acid identities at the five variable sites. We used unpaired Welch's t-tests between each variant and wtMxA to evaluate statistical significance (*p-value < 0.05).

Fig. S2.

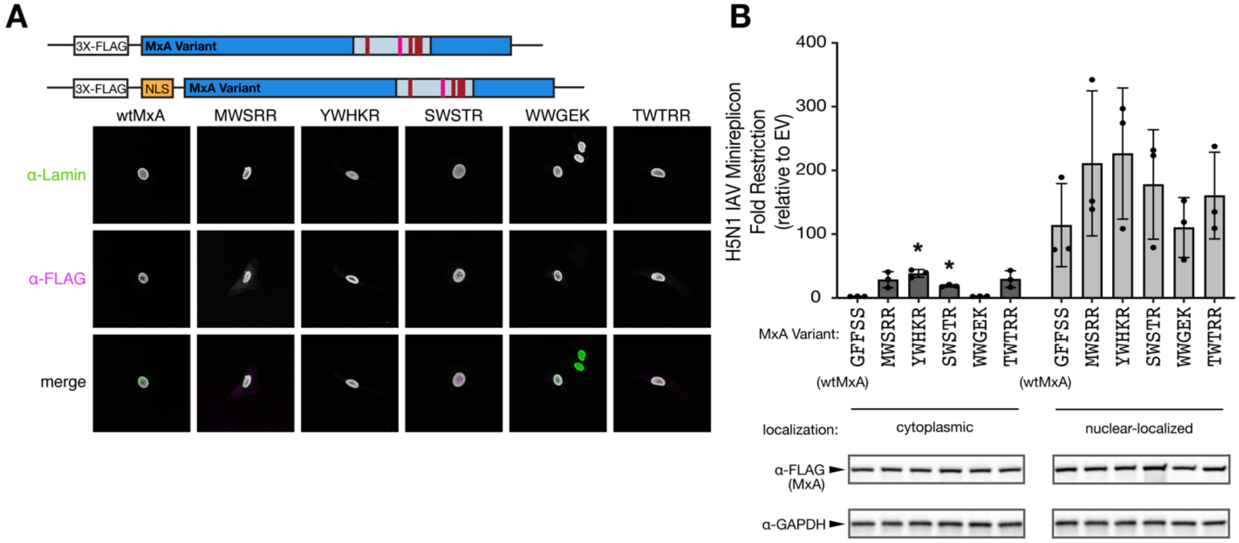

Figure S2: Nuclear localization further enhances H5N1 super-restriction. (A) The SV40 large T antigen nuclear localization signal (NLS) PKKKRKV was cloned into the N-termini of MxA variants between a 3X-FLAG tag and the MxA gene. NLS-tagged variants were transfected into HeLa cells and imaged in the same manner as described in Fig. 2A. Images are projections of the z-stacks spanning the height of the cells. **(B)** The five super-restrictor variants, as well as wtMxA, with and without an N-terminal NLS were assayed for their H5N1 restriction relative to an empty vector control in the minireplicon assay. Their expression levels were also tested by Western blotting. Unpaired Welch's t-tests were performed between restriction levels of cytoplasmic variants and wtMxA as well as between NLS-tagged variants and NLS-wtMxA (* $p < 0.05$).

Fig. S3.

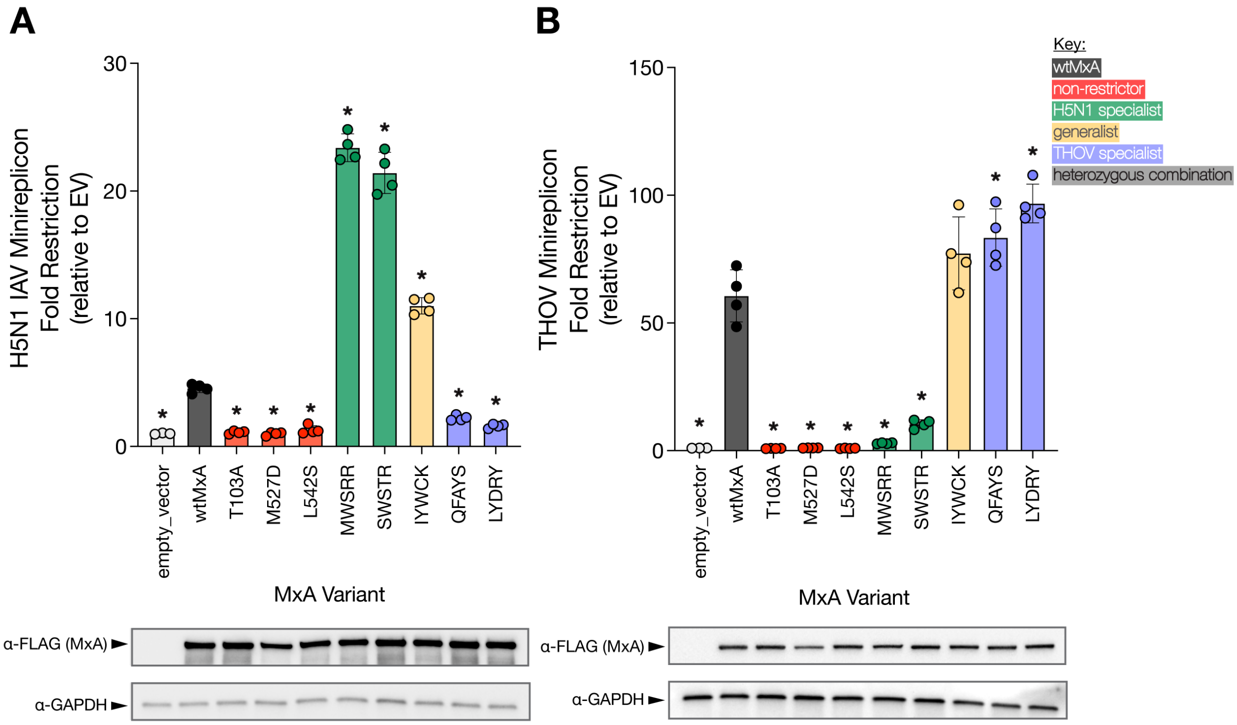

Figure S3: 'Generalist' and 'specialist' MxA variants restriction of H5N1 and THOV. We retested H5N1 **(A)** or THOV **(B)** restriction by wtMxA, non-restricting controls, specialist, and generalist MxA variants relative to an empty vector control based on a minireplicon assay; data is represented on a linear scale. The total amount of empty vector or MxA variant per condition is 100ng per well for H5N1 assay and 50 ng per well for the THOV assay. Unpaired Welch's t-tests were performed between restriction levels of cytoplasmic variants and wtMxA as well as between NLS-tagged variants and NLS-wtMxA (* p< 0.05).

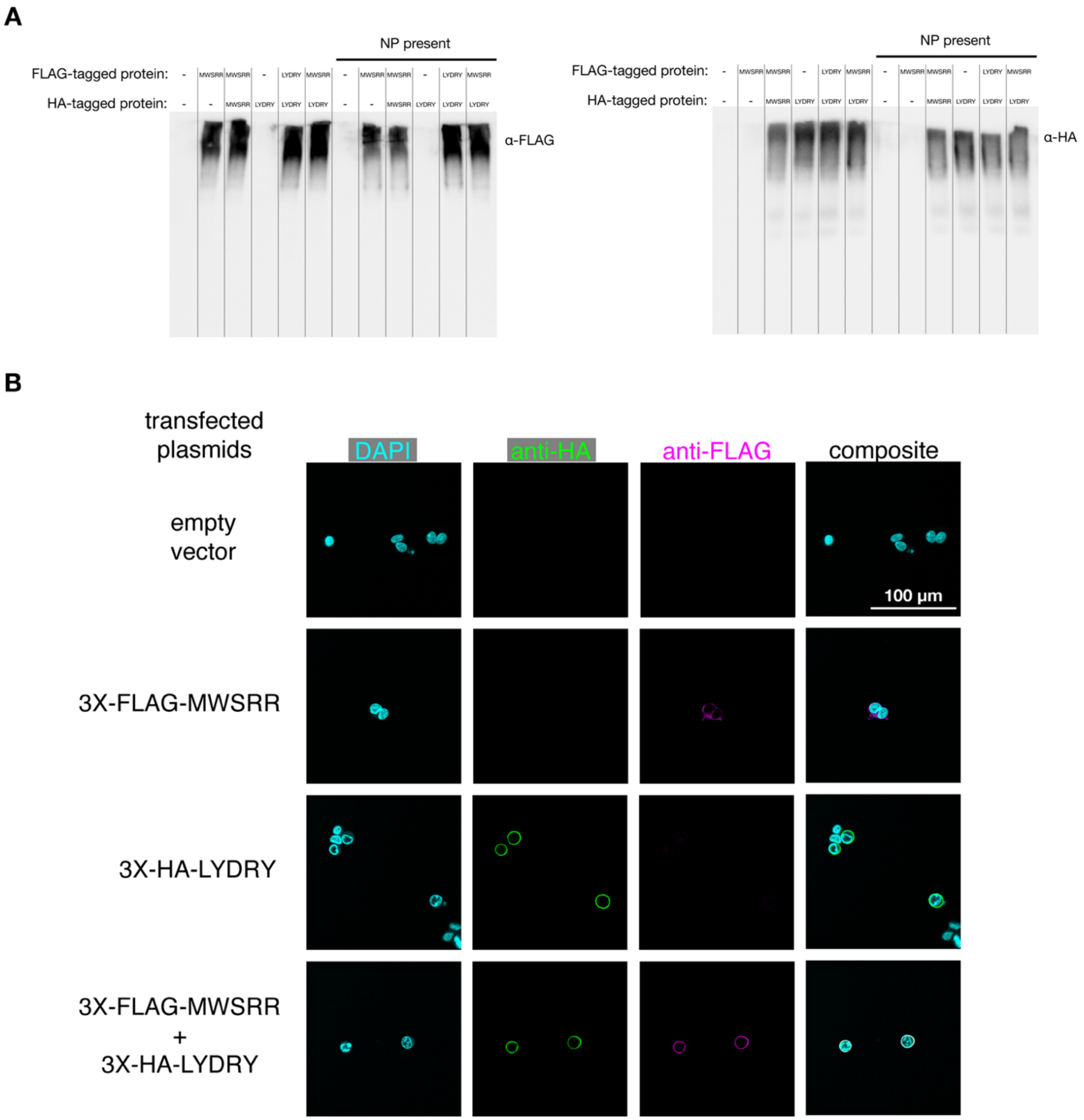

Figure S4: Heterozygous alleles oligomerize similarly and are co-expressed in individual HEK293T cells. HEK293T cells were transfected with combinations of MxA variants possessing either an N-terminal 3X-FLAG tag or 3X-HA tag and tested for oligomerization by native PAGE gel followed by Western blotting **(A)** and for cellular localization by immunofluorescence microscopy **(B).** (A) Western blotting revealed high-order oligomers of all variants and variant combinations that stained at a molecular weight range of ~150kD - >250kD. Blots with the colorometric image showing protein standards overlayed can be found in the Supplemental Raw Images. **(B)** DAPI staining is indicated in cyan, anti-HA staining is indicated in green, and anti-FLAG staining is indicated in magenta. The overlap of any two stains is indicated in white. Images are of the z-slice at which DAPI staining was brightest. Note the significantly smaller cytoplasmic area in these cells compared to those in Figure 1E. This is due to the morphological differences between HeLa cells (Fig. 1E) HEK293T cells.

Supplementary tables S1 to S15. (separate file)
Table S1. Raw luciferase signal readings for H5N1 IAV minireplicon assay with MxA variants in which amino acid position 561 is mutated to a non-aromatic amino acid. See supplemental figure S1.

Table S2. Raw luciferase signal readings for H5N1 IAV minireplicon assay screen of restriction by 194 MxA loop L4 variants. See Figure 1B.

Table S3. Raw luciferase signal readings for H5N1 IAV minireplicon assay with top super-restrictor variants identified in the screen from Figure 1B. See Figure 1C.

Table S4. Raw luciferase signal readings for H5N1 IAV minireplicon assay with four validated super-restrictors at varying doses of plasmid input. See Figure 1D.

Table S5. Raw luciferase signal readings for H5N1 IAV minireplicon assay with MxA loop L4 variants both with and without an N-terminal SV40 NLS peptide signal. See supplemental figure S2B.

Table S6. Raw luciferase signal readings for H5N1 IAV minireplicon assay with MxA loop L4 variants in which each residue of SWSTR was individually reverted to their wildtype residues (GFFSS) and vice versa from wildtype to SWSTR. See Figure 2C.

Table S7. Raw luciferase signal readings for H5N1 IAV minireplicon assay in which MxA variants with two amino acids of wtMxA were simultaneously mutated to test for epistasis in super-restriction. See Figure 2D.

Table S8. Raw luciferase signal readings for minireplicon assays testing a panel of 52 MxA loop L4 variants against both H5N1 IAV and THOV. See Figure 3A.

Table S9. Raw luciferase signal readings for minireplicon assay testing three MxA loop L4 variant backbones with each of the three aromatic amino acids (F/W/Y) at position 561 against both H5N1 IAV and THOV. See Figure 3B.

Table S10. Raw luciferase signal readings for the H5N1 IAV minireplicon assay with wtMxA, non-restricting controls, specialist, and generalist MxA variants. See supplemental figure S3A.

Table S11. Raw luciferase signal readings for the THOV minireplicon assay with wtMxA, non-restricting controls, specialist, and generalist MxA variants. See supplemental figure S3B.

Table S12. Raw luciferase signal readings for the H5N1 IAV minireplicon assay with equimolar combinations of MxA variants with wtMxA. See Figure 4A.

Table S13. Raw luciferase signal readings for the THOV minireplicon assay with equimolar combinations of MxA variants with wtMxA. See Figure 4B.

Table S14. Raw luciferase signal readings for H5N1 IAV minireplicon assay with the H5N1 specialist MWSRR, THOV specialist LYDRY, or their equimolar combination. See Figure 4C.

Table S15. Raw luciferase signal readings for THOV minireplicon assay with the H5N1 specialist MWSRR, THOV specialist LYDRY, or their equimolar combination. See Figure 4D.

Supplementary data S1. (separate file)

Data S1. Full Western Blot images for Westerns reported in text, main figures, and supplementary figures. Images were captured on a BioRad Gel Doc after incubation with HRP substrate (see Methods). Each raw image is labeled with its associated main text or supplemental figure, sample identifiers, and antibodies used.
