# Supplementary material for: Heterozygous and generalist MxA super-restrictors overcome breadth-specificity trade-offs in antiviral restriction
Source: Sci Adv. 2025 May 2;11(18):eadu0062. doi: 10.1126/sciadv.adu0062 (PMC12047444; doi:10.1126/sciadv.adu0062)
Supplement: Supplementary file 3 — Data S1 [file sciadv.adu0062_data_s1.zip › adu0062_data_s1.pdf]

Figure 1C

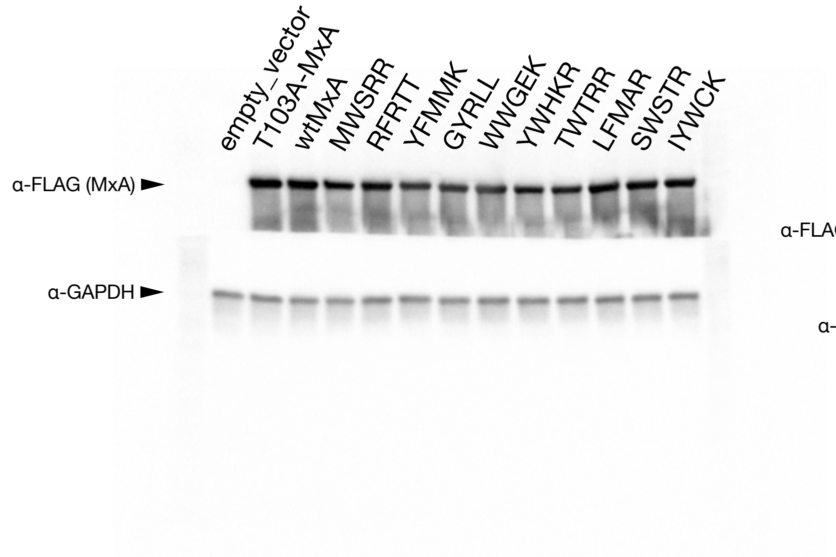

Figure 1D

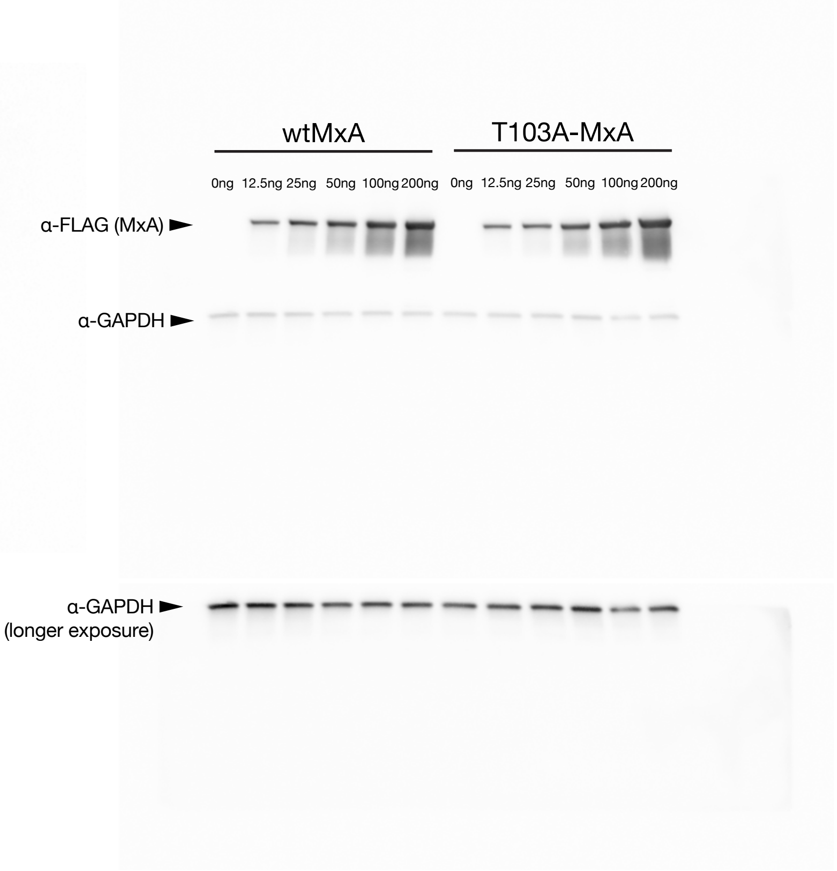

Figure 1D

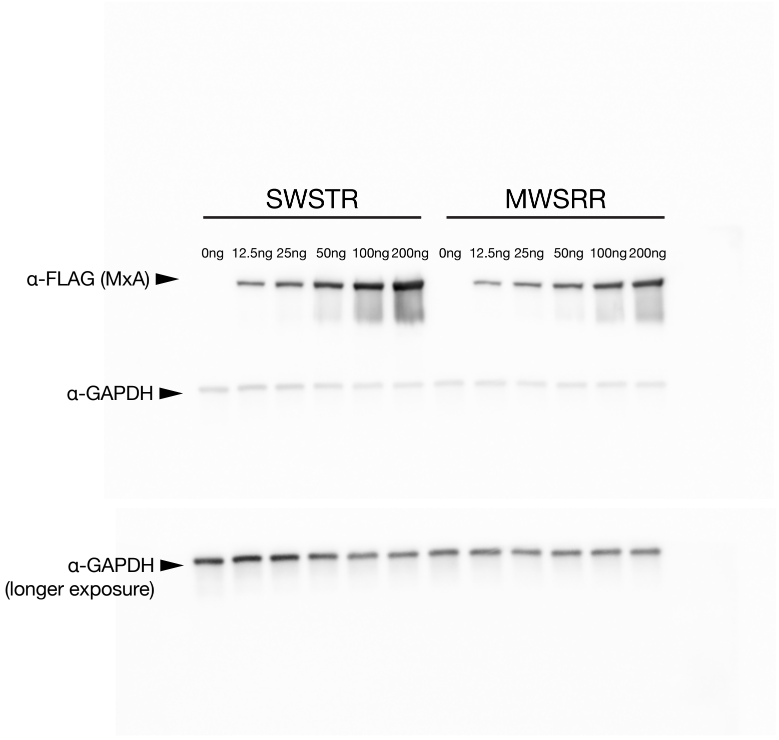

Figure 1D

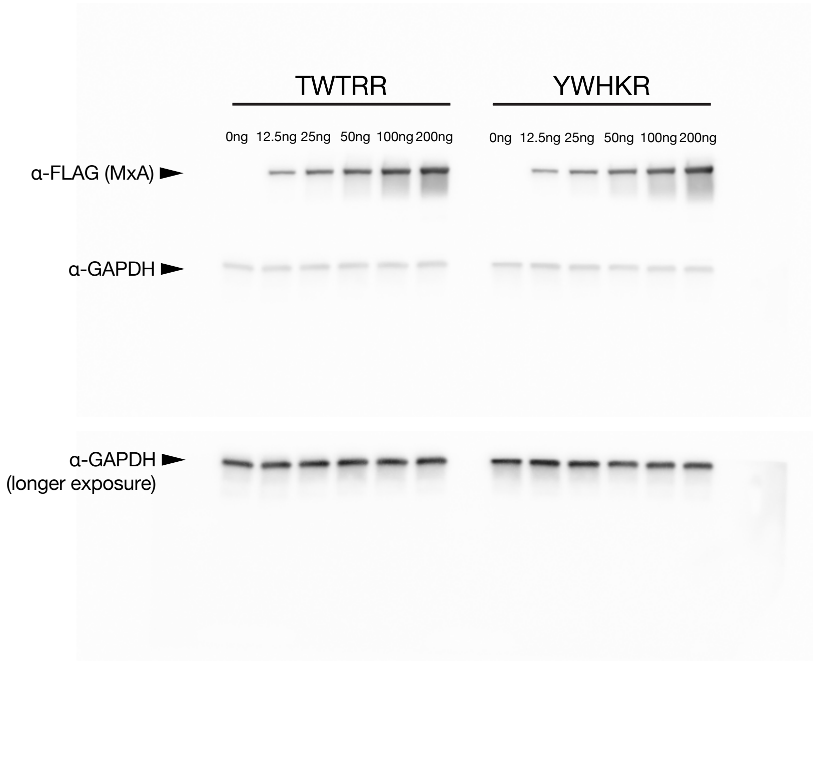

Raw Images

Figure S2B

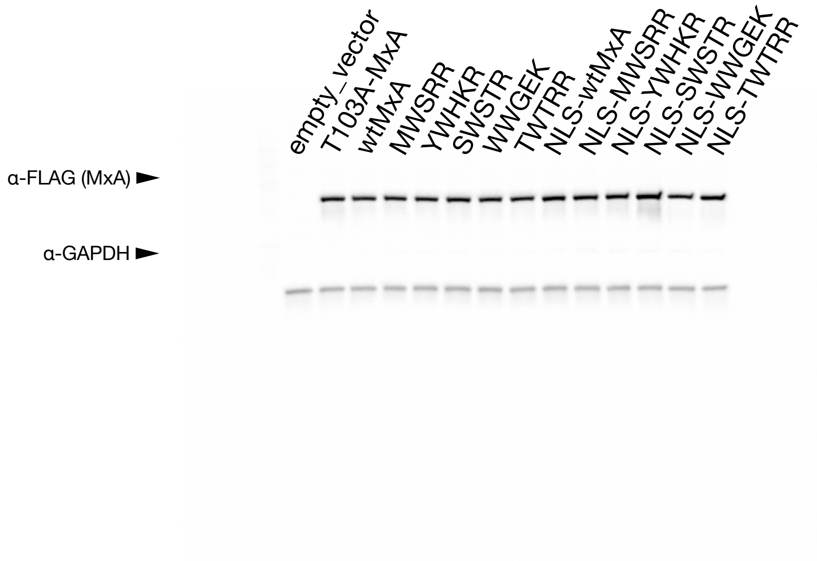

Figure 2C - left

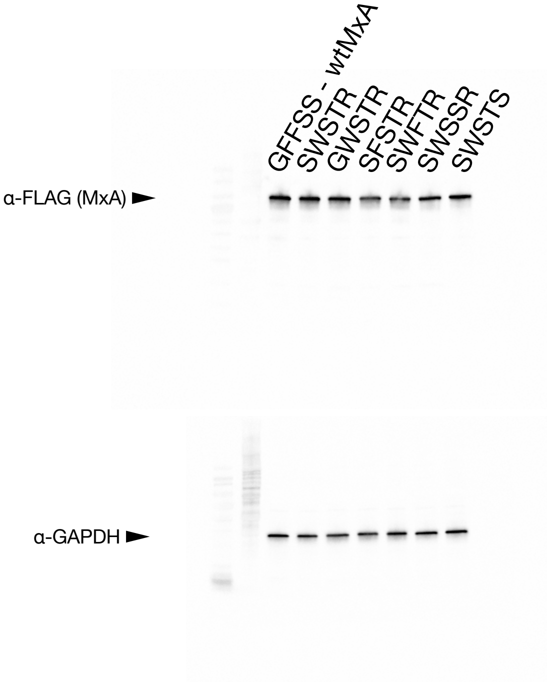

Figure 2C - right

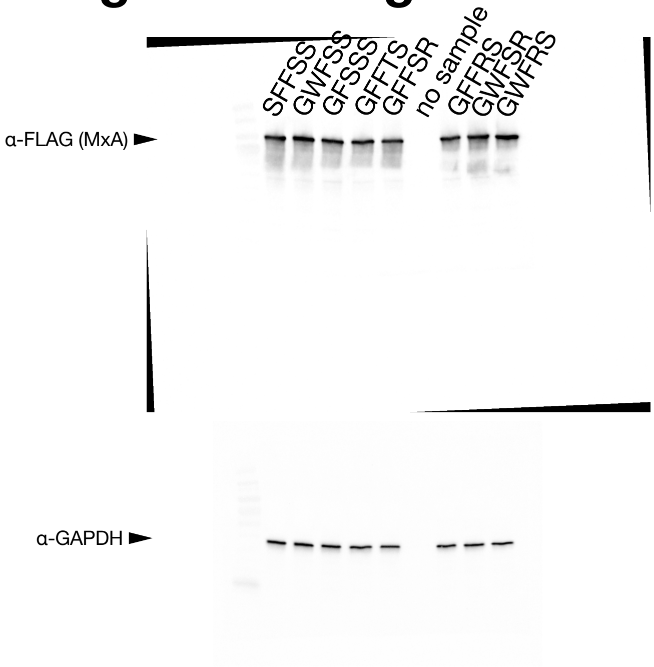

Figure 2D

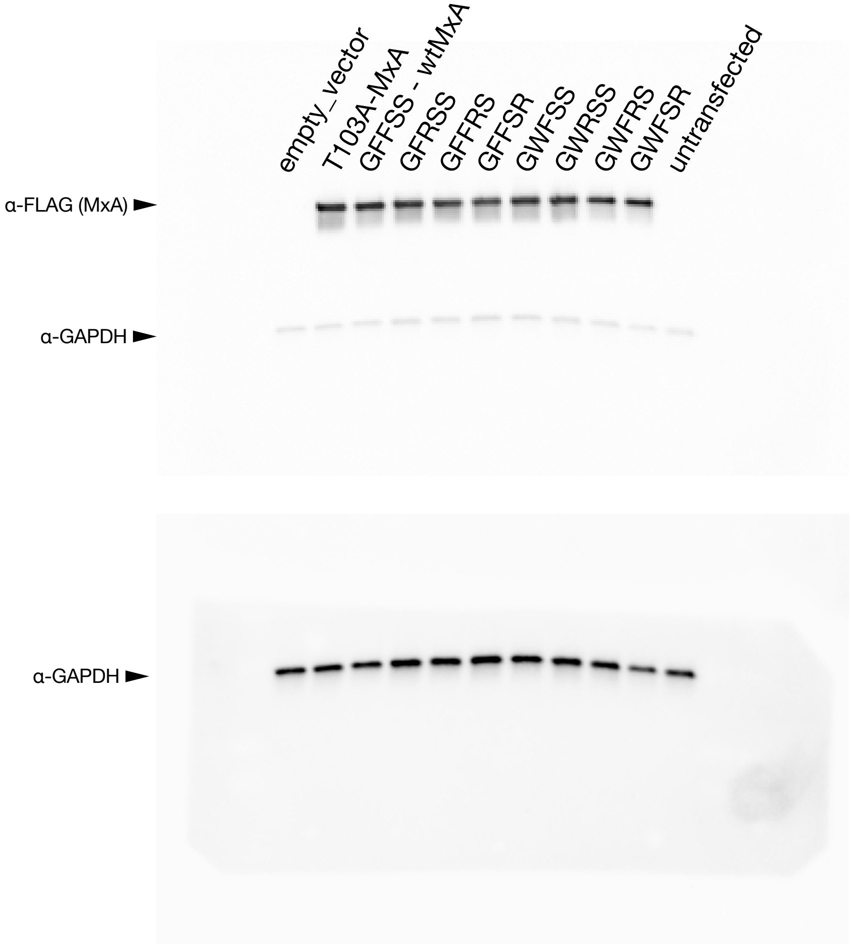

Raw Images

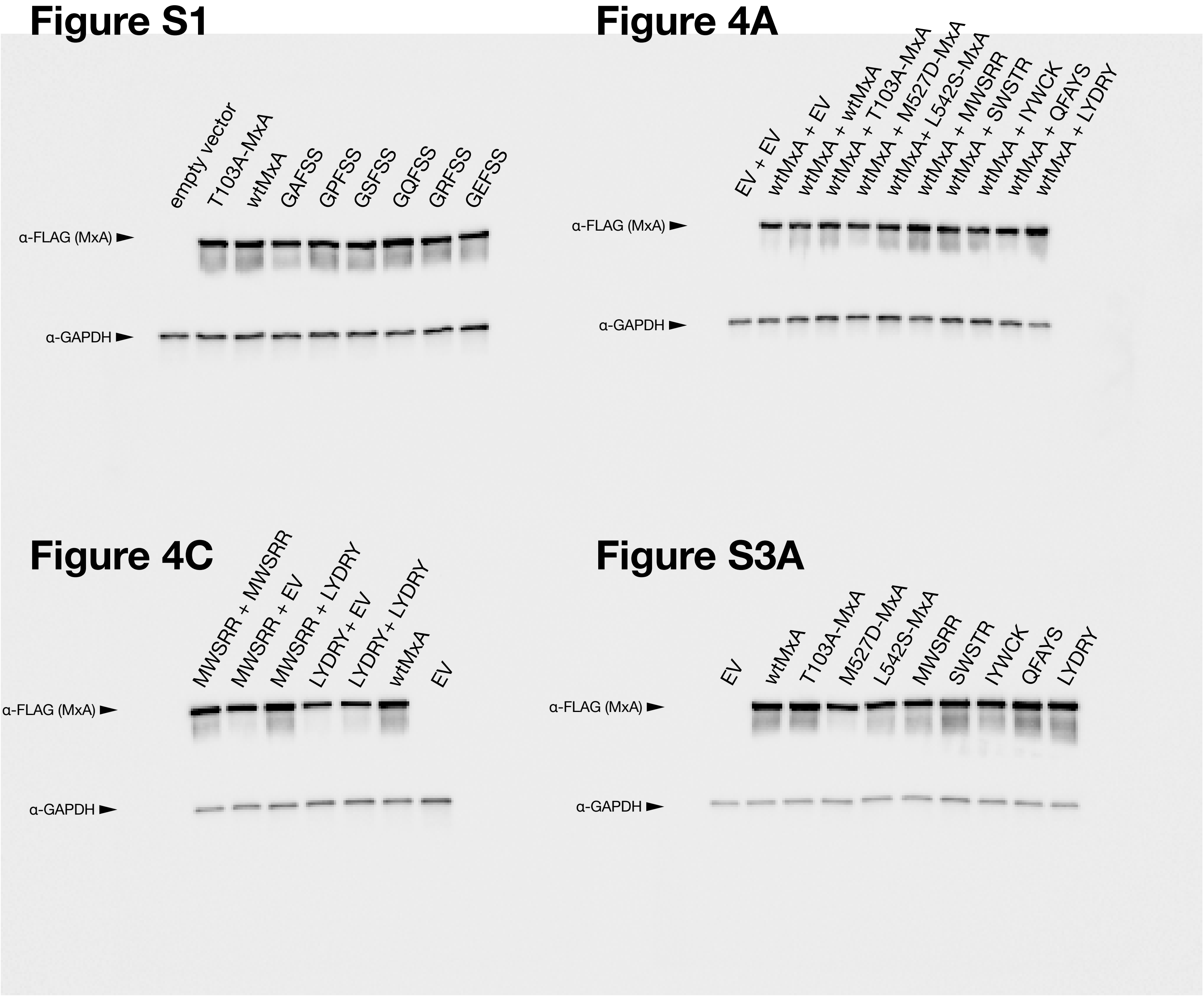
Figure S1
empty vector
T103A-MxA
wtMxA
GAFSS
GPFSS
GSFSS
GQFSS
GRFSS
GEFSS
α-FLAG (MxA)
α-GAPDH
Figure 4A
EV + EV
wtMxA + EV
wtMxA + wtMxA
wtMxA + T103A-MxA
wtMxA + M527D-MxA
wtMxA+ L542S-MxA
wtMxA + MWSRR
wtMxA + SWSTR
wtMxA + IYWCK
wtMxA + QFAYS
wtMxA + LYDRY
α-FLAG (MxA)
α-GAPDH
Figure 4C
MWSRR + MWSRR
MWSRR + EV
MWSRR + LYDRY
LYDRY+ EV
LYDRY+ LYDRY
wtMxA
EV
α-FLAG (MxA)
α-GAPDH
Figure S3A
EV
wtMxA
T103A-MxA
M527D-MxA
L542S-MxA
MWSRR
SWSTR
IYWCK
QFAYS
LYDRY
α-FLAG (MxA)
α-GAPDH

Raw Images

Figure 4B

Figure 4D

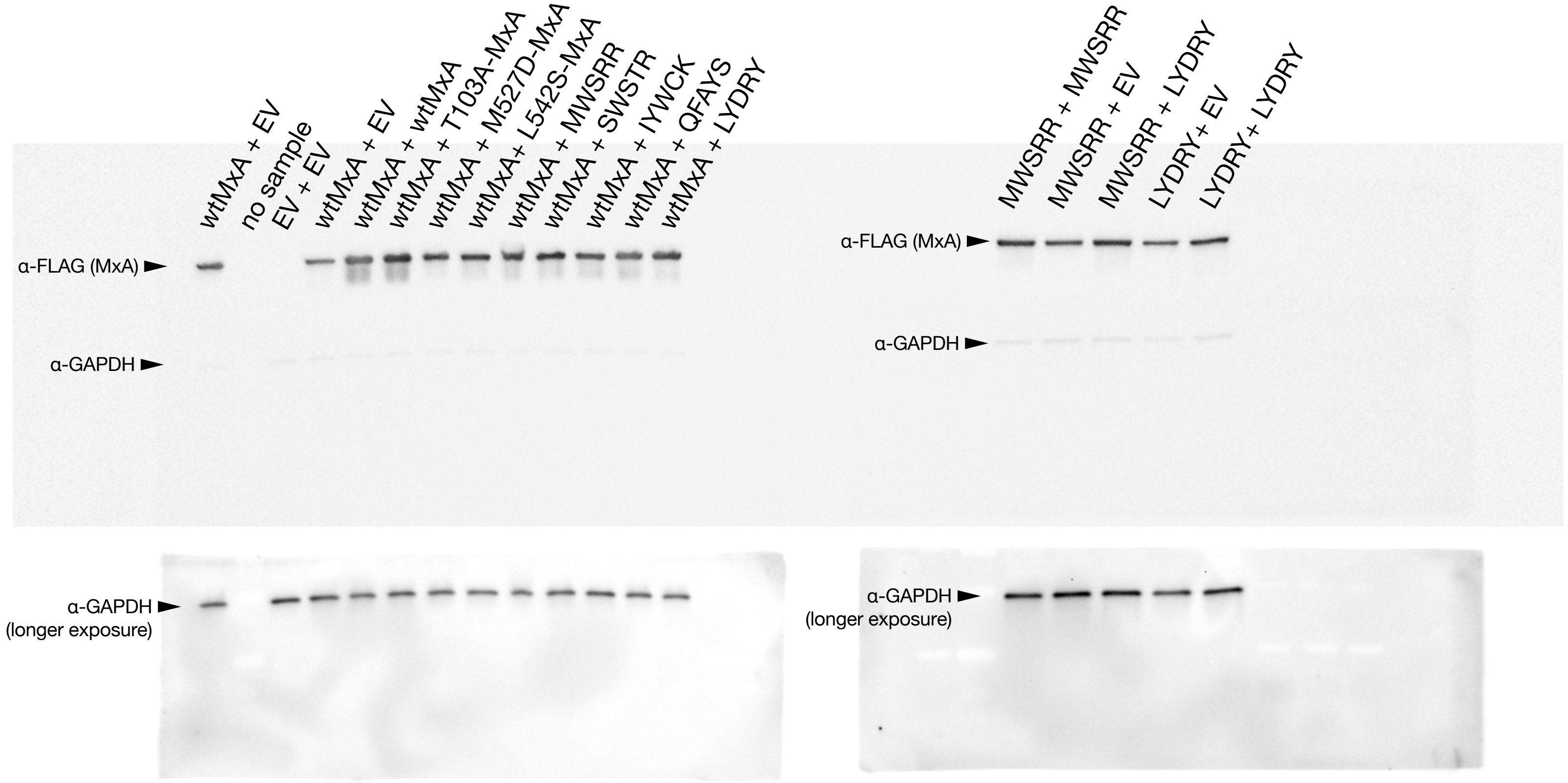

Figure S3B

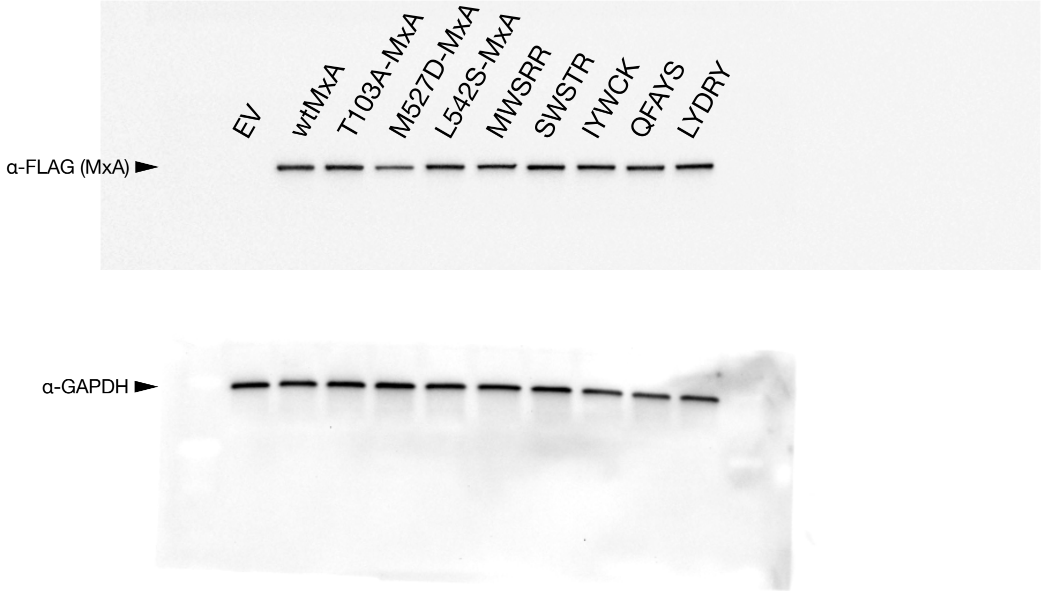

Raw Images

Figure 4E

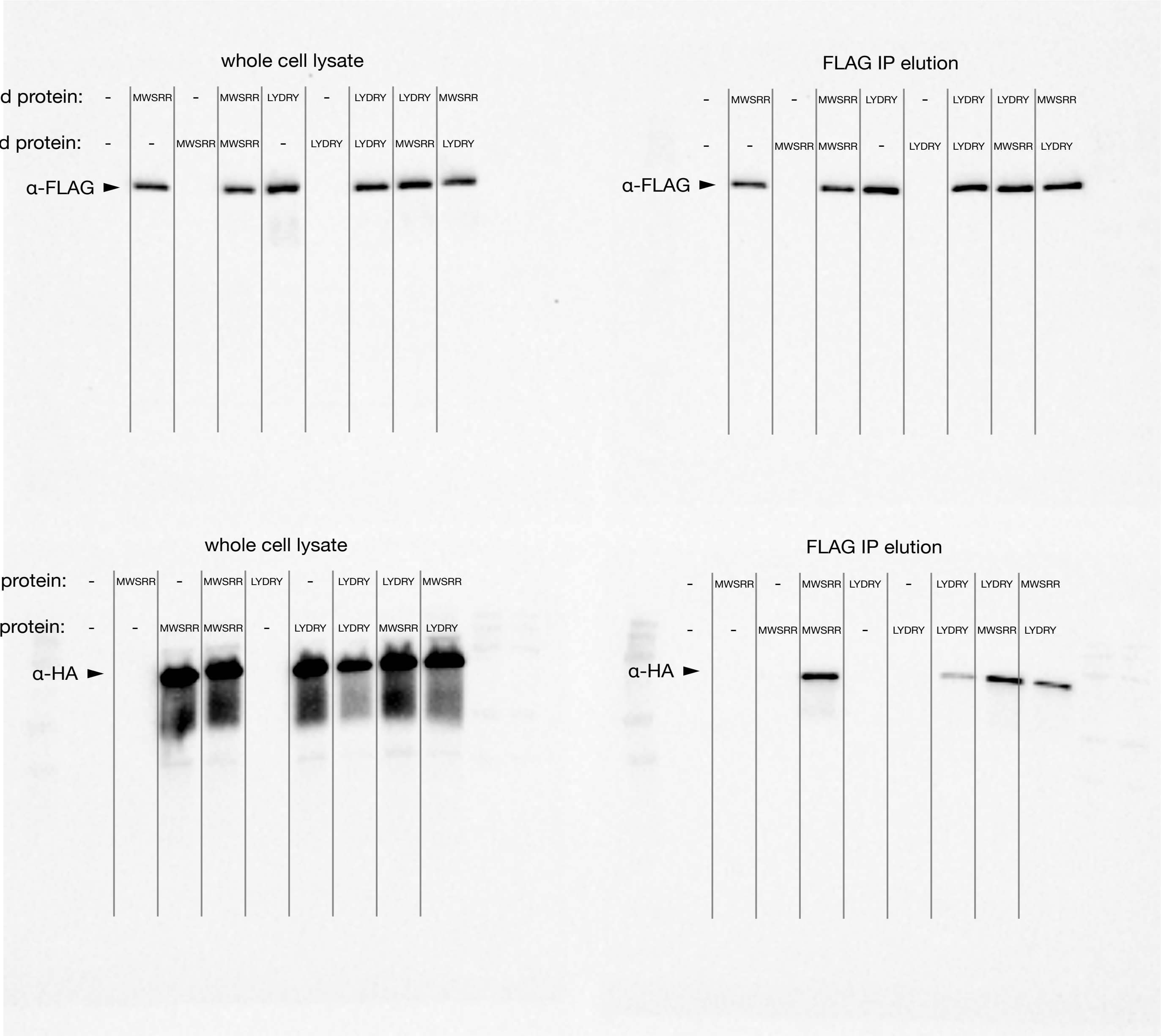

Figure S4A

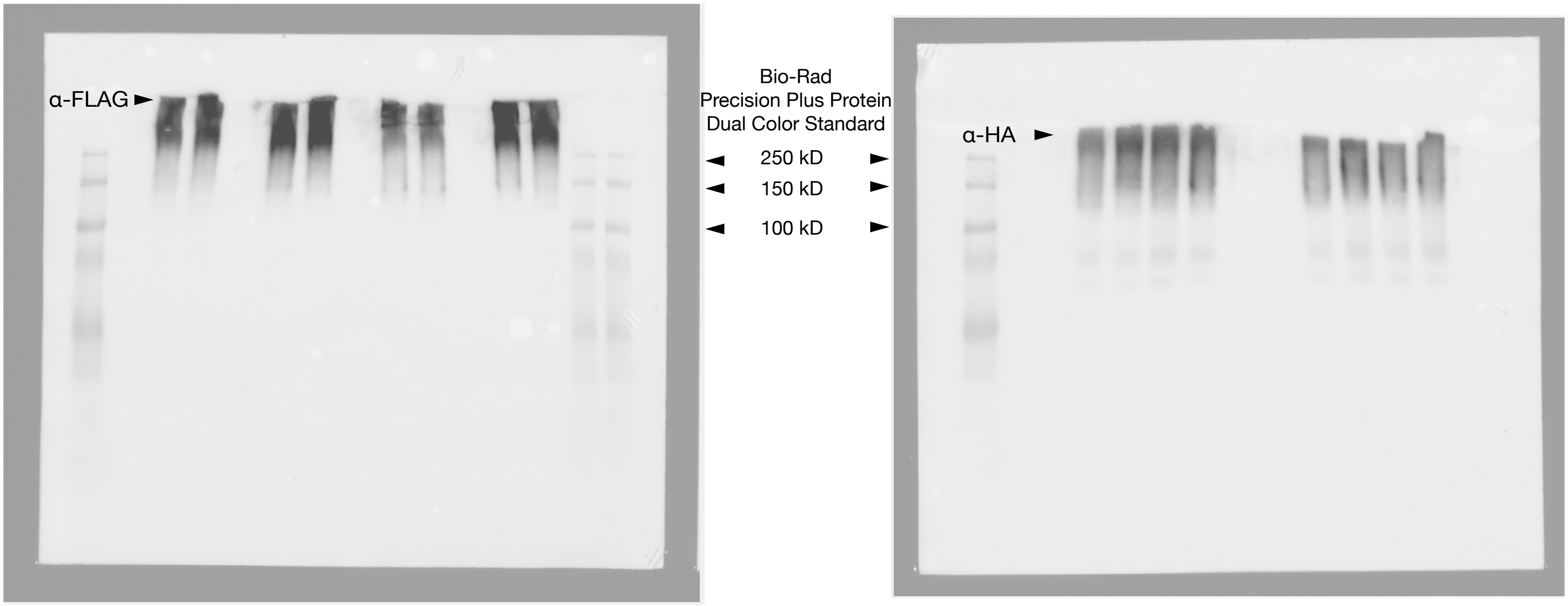

Raw Images
